# Impact of COVID-19 on Gynecologic Oncology Wait Times: A Mystery Caller Study

**DOI:** 10.7759/cureus.72328

**Published:** 2024-10-24

**Authors:** Mackenzie E Lemieux, Kati Turner, Josh Durfee, Spyridon Mastroyannis, Tyler Muffly

**Affiliations:** 1 Obstetrics and Gynecology, Washington University School of Medicine, Missouri, USA; 2 Obstetrics and Gynecology, Denver Health and Hospitals, Denver, USA; 3 Obstetrics and Gynecology, Denver Health Medical, Denver, USA; 4 Obstetrics and Gynecology, Denver Health Hospital Authority, Denver, USA

**Keywords:** access to care, covid-19, gynecologic oncology, mystery caller study, wait times

## Abstract

Objective: Despite increasing wait times for oncologic care in the US, research has yet to examine the impact of COVID-19 on wait times to first appointments for gynecologic oncology patients. We sought to audit mean wait times, during and after the height of the pandemic, for an outpatient appointment with a gynecologic oncologist in the US.

Methods: Office phone numbers were identified from the searchable Society for Gynecologic Oncology specialist patient-facing database. Using a “mystery caller” study approach, each unique phone number was called in 2020 and 2023. The caller asked for the soonest appointment available for her mother, who was recently found to have a 10 cm pelvic mass. The date of the soonest appointment and physician and office demographics were collected.

Results: A total of 222 gynecologic oncology practices were called across 45 states and the District of Columbia. There was no difference in wait time post-COVID-19, highlighting an undescribed resilience in the face of unprecedented healthcare system stress. However, we also identified three major barriers to appointment scheduling including incorrect contact information in patient-facing databases, unanswered phones, and mandatory physician referrals prior to appointment scheduling.

Conclusions: Understanding factors influencing appointment wait times is essential to mitigating harm in oncologic care. Ours is the first nationwide audit of COVID-19’s impact on barriers to gynecologic oncology care. While we highlight a surprising lack of increase in wait times between 2020 and 2023, we also identify actionable barriers to care such as updating public patient-facing information online.

## Introduction

Long wait times for oncologic care lead to lowered survival rates, lower quality of life, and greater frustration with the healthcare system, constituting a significant issue worldwide [1]. In the US specifically, wait times for oncologic care have been increasing in recent decades [1,2]. Among patients seeking cancer consultation and treatment, long wait times can compromise a patient’s survival [3]. Historically, disenfranchised patients, including those of low socioeconomic status, non-white race, older adults, and disabled patients, are disproportionately impacted by long wait times, leading to poorer outcomes [1]. Therefore, minimizing the elapsed time between observation of symptoms and initiation of treatment represents a modifiable target to improve oncologic outcomes.

Within the field of gynecologic oncology, delays in obtaining a diagnosis typically occur at one of three junctures: (1) the length of time to first seeing a physician, (2) the length of time to seeing a subspecialist, and (3) the length of time to diagnosis [4]. While research has explored how patient education and primary care triage can improve delays in patient intervals, little research has focused on the delays in the length of time to see a subspecialist. Studies performed outside the US have reported various lengths of time for this interval: zero to 22 days in the UK, one day in Denmark, and 19 days in Manitoba, Canada, respectively [4,5]. However, these studies took place in systems with universal healthcare, meaning these results were comparably easier to obtain than in the US. Median delays in seeing a subspecialist have yet to be thoroughly or systematically investigated in the US concerning gynecologic oncology care. 

One way that audits of healthcare system performance can be conducted is through “mystery caller” studies [6]. This approach uniquely assesses the patient experience, access to care, and wait times from a patient perspective. Through mystery caller studies, differences in wait times for gynecologic subspecialists for patients with public versus private health insurance have been elucidated [7,8]. Here, we sought to use a mystery caller approach to evaluate delays in the primary care interval for patients seeking specialized gynecologic oncology care during the time surrounding the COVID-19 pandemic.

During the height of the pandemic, patients with worse oncologic status were prioritized based on guidelines released by the Society of Gynecologic Oncology (SGO) [9]. Interestingly, research from one institution showed that overall wait times for endometrial cancer and precancer surgery increased by up to 75% between 2019 and 2021 [10]. However, the lasting impact on wait time since 2020 has not been assessed. In the wake of the emergency phase of the COVID-19 pandemic, researchers have yet to assess the lasting impacts of this significant health system stressor on delays in gynecologic oncology care. Here, we sought to understand the ongoing effects of the COVID-19 pandemic on wait times for first appointments with a gynecologic oncologist in the United States. We applied a mystery caller approach to compare Fall 2020 (early in the pandemic) and Fall 2023 (post-emergency phase) wait times in gynecologic oncology care. We hypothesized that wait times would be increased in the post-pandemic era as the system catches up from the backlog. 

## Materials and methods

Call list collation

A list of public provider National Provider Identifiers (NPIs) was obtained from the Centers for Medicare and Medicaid Services (cms.gov) website. The list included data on provider specialty and subspecialty, the date each provider began practicing, and the state of practice. Data were downloaded and limited to providers who started practicing no later than December 31, 2019. The Denver Health Office of Research approved the protocol, and this study was deemed non-human subject research by the Colorado Multiple Institutional Review Board.

Study data were collected and managed using Research Electronic Data Capture (REDCap) electronic data capture tools hosted at the Denver Health Office of Research [11,12]. REDCap is a secure, web-based software platform designed to support data capture for research studies, providing 1) an intuitive interface for validated data capture; 2) audit trails for tracking data manipulation and export procedures; 3) automated export procedures for seamless data downloads to common statistical packages; and 4) procedures for data integration and interoperability with external sources.

Data cleaning and preparation of the list of physician phone numbers were completed in Exploratory.io (version 6.2.2.1: Exploratory, Inc., Redwood City, US). Providers from the Centers for Medicare and Medicaid Services list were removed from the data if they were listed as “retired,” practicing in Canada, or did not have an active or valid board certification. Finally, the data were limited to providers with “gynecologic oncology” listed as their taxonomy code.

After this cleaning process, the data were then sampled to identify up to six providers from each state, with two providers from each practice start year category. If two providers were unavailable from a particular state and start year category, the one available provider was included. If no providers were available from the state and start year category, the data were not supplemented with providers from other start year categories.

The SGO maintains a searchable online patient-facing database of actively practicing subspecialists in all professions related to gynecologic cancer (specialist.sgo.org). With “gynecologic oncologist” selected in the dropdown list for the profession, each name in the sampled dataset was searched to identify a phone number to contact the physician.

Data gathering

The first data-gathering episode occurred from October 19, 2020, to November 9, 2020, while the second occurred from September 8, 2023, to October 17, 2023. During both episodes, one female author called gynecologic oncology offices during regular business hours.

During the call, the caller requested the soonest available appointment for her mother using the script detailed below. The patient's name, age, and chief complaint remained the same throughout both episodes of data gathering. If asked, the patient said to have Blue Cross/Blue Shield insurance. A call transfer was defined as indicating another selection on the electronic prompt OR being transferred by a human.

“Hi! I’m calling on behalf of my mom. She was recently seen at the ER with abdominal pain, and they found a 10 cm mass in her pelvis. I’ve been calling oncologists in the area to see when she might be able to be seen. Do you know when your first appointment with a physician might be?”

Gynecologic oncology offices were unaware that research was being conducted until after the calls were completed. Because the offices were not informed of their participation during the study, we applied ethical safeguards, including (1) telephone calls to the office staff were made in a routine manner that attempted to minimize time spent on the telephone, (2) no appointments were scheduled, (3) after the calls were completed, all offices received debriefing letters that detailed the nature of the study, and they were encouraged to contact the research team with any questions or concerns.

If the phone number listed did not correspond to the expected clinical office, but prompts could be successfully followed via the electronic answering system, or the individual answering the phone could transfer the caller to the appropriate office, the caller proceeded with trying to establish the next available appointment date with the office. If the answering individual provided the phone number for the clinical office, this number was recorded but was not called to allow the data to reflect the integrity of the SGO provider contact database. If the hold time exceeded five minutes or there was no answer, an attempt was made to contact the office again on the following business day. On the second call attempt, if the hold time exceeded five minutes or there was no answer, no further effort was made to contact the office.

**Figure 1:**
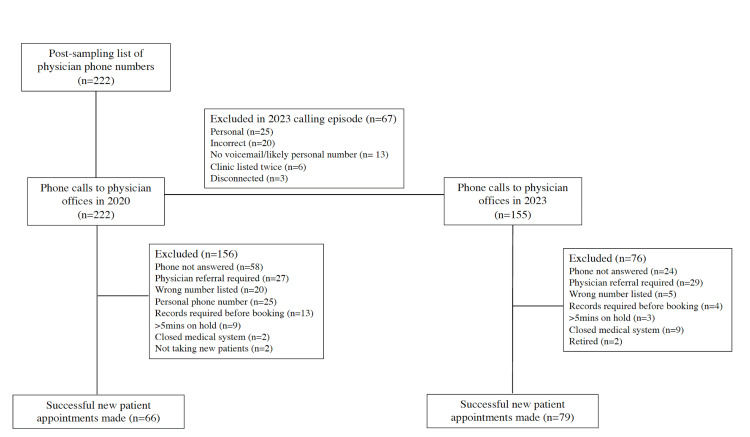
Flow chart depicting the reasons for physician exclusion in the wait time calculation for each calling episode (2020 and 2023). Each box represents a specific step and actions taken, while each line represents the flow of the process. All successful new patient appointments were included in the wait time calculation

After contacting each office on the list, offices were excluded from analysis if (1) the number contacted did not correspond to the expected office or specialty (i.e., an academic office or clinical practice other than gynecologic oncology), (2) a physician referral was required prior to scheduling, (3) medical records were required before scheduling, (4) the phone number was a physician’s personal phone, (5) the call went to voicemail, (6) there was no answer or a busy signal on repeat calls, (7) greater than five minutes were spent on hold, (8) the practice was part of a closed health system (e.g., Kaiser, military hospital), or (9) the practice was not accepting new patients (Figure 1).

Office phone numbers that were deemed incorrect or disconnected in 2020 were automatically excluded from the 2023 data-gathering period. Otherwise, all other criteria used in the 2020 data-gathering stage were replicated during the 2023 data-gathering stage. Information was also collected from offices that could not provide an appointment to ascertain why a prospective patient may be unable to receive care at these locations.

For information not available in the SGO database, the authors (KT and ML) conducted an independent online search. Physician age was determined by searching each physician's name at healthgrades.com [13]. Practice type was defined as academic if the physician was affiliated with an academic center, determined through a Google search. If the physician was not affiliated with an academic center, their practice type was defined as private. Data on which physicians worked at institutions was found on the list of “National Cancer Institute -Designated Cancer Center” [14]. 

Data analysis and statistical testing

Initial data preparation and analyses were done in Exploratory.io (Version 9.4.1: Exploratory, Inc., Redwood City, US). Data modeling and further analyses were performed using SAS Enterprise Guide Software version 8.3 (SAS Institute Inc., Cary, US). A linear regression model was used to determine the number of business days until an appointment could be scheduled. This model adjusted for differences in National Cancer Institutes (NCI) affiliation of the clinic, physicians’ gender, insurance, and employed generalized estimating equations (GEE) to account for the within-subject correlation of repeated measures by individual clinics called in both time periods. T-tests and chi-square tests were used for continuous and categorical bivariate comparisons, respectively.

## Results

An initial call list containing 222 gynecologic oncologists with phone numbers was created in 2020 via the sampling method described in the methods. As shown in Figure 1, all of the gynecologic oncologists on the list were called in 2020, and a subset was called in 2023, for a total of 377 calls over the study period. A preliminary analysis pooled all 377 calls across the 2020 and 2023 calling cycles and compared the characteristics of gynecologic oncologists and their associated offices that provided appointment dates during the mystery call (145 total offices provided an appointment date) versus those that did not provide appointment dates (232 did not provide appointment dates) (Table 1, Figure 1). Overall, there was no difference in mean age of the physician, first year of physician practice, NCI affiliation status, or American College of Obstetricians and Gynecologists (ACOG) district across offices that were able to offer appointment dates versus those that did not. Private offices were significantly more likely to offer appointments than academic offices (56.6% private versus 43.4% academic; p=0.04).

**Table 1:** Characteristics of gynecologic oncology offices by appointment offered. Table comparing the characteristics of gynecologic oncologists and associated offices that were included in the wait time calculation (thus offered an appointment date) versus those that were excluded from the wait time calculation NCI: National Cancer Institutes; ACOG: American College of Obstetricians and Gynecologists

Characteristic	Offered appointment		Significance
	No	Yes	P-value
	(n=232)	(n=145)	
Age (years)			
mean (SD)	58 (11)	60 (10)	p=0.14
First year of physician practice N(%)			
1954-1976	12 (5.2%)	7 (4.8%)	p=0.49
1977-1997	99 (42.7%)	71 (49.0%)	
1998-2019	121 (52.1%)	67 (46.2%)	
Practice type N(%)			
Academic	126 (54.3%)	63 (43.4%)	p=0.04
Private	106 (45.7%)	82 (56.6%)	
NCI affiliated institution N(%)			
Yes	71 (30.6%)	36 (24.8%)	p=0.23
No	161 (69.4%)	109 (75.2%)	
ACOG District N(%)			
District I (Atlantic Provinces, CT, ME, MA, RI, VT)	22 (9.5%)	10 (6.9%)	p=0.52
District II (NY)	9 (3.9%)	4 (2.8%)	
District III (DE, NJ, PA)	17 (7.3%)	6 (4.1%)	
District IV (DC, GA, MD, NC, SC, VA, WV)	38 (16.4%)	20 (13.8%)	
District V (IN, KY, OH, MI)	22 (9.5%)	17 (11.7%)	
District VI (IL, IA, MN, NE, ND, SD, WI)	27 (11.6%)	14 (9.6%)	
District VII (AL, AR, KS, LA, MS, MO, OK, TN)	38 (16.4%)	27 (18.6%)	
District VIII (AK, AZ, CO, HI, ID, MT, NV, NM, OR, UT, WA, WY)	35 (15.1%)	32 (22.1%)	
District IX (CA)	10 (4.3%)	4 (2.8%)	
District XI (TX)	12 (5.2%)	4 (2.8%)	
District XII (FL)	2 (0.9%)	7 (4.8%)	

Further analyses were limited to the 145 offices that were able to provide appointments to focus on the questions surrounding wait time. Differences in characteristics of gynecologic oncologists were assessed by year (2020, N=66 versus 2023, N=79 from Table 2). The mean age of gynecologic oncologists was 59 (SD±10) in 2020 and 60 (SD±10) in 2023 (p=0.40), although 26 physicians of the original 222 did not have an age listed on healthgrades.com. More than half of the gynecologic oncologists in our study started practicing before 1998 (51.5% in 2020 and 55.7% in 2023). More male physicians were available than female physicians both in 2020 (65.1% male versus 34.8% female) and 2023 (53.2% male versus 46.8% female); however, the difference was not significant (p=0.14). The proportion of available female physicians increased by 12% from 2020 to 2023 (34.8% to 46.8%).

**Table 2:** Characteristics of gynecologic oncology offices that offered appointments by year. Table comparing the characteristics of gynecologic oncologists and associated offices that offered appointments across 2020 and 2023 NCI: National Cancer Institutes; ACOG: American College of Obstetricians and Gynecologists

Characteristic	Year		Significance
	2020	2023	P-value
	(N=66)	(N=79)	
Age (years)			
mean (SD)	59 (10)	60 (10)	p=0.4
Available physician gender N(%)*			
Male	43 (65.1%)	42 (53.2%)	p=0.14
Female	23 (34.8%)	37 (46.8%)	
First year of physician practice N(%)			
1954-1976	3 (4.5%)	4 (5.1%)	p=0.88
1977-1997	31 (47.0%)	40 (50.6%)	
1998-2019	32 (48.5%)	35 (44.3%)	
Practice type N(%)			
Academic	23 (34.8%)	40 (50.6%)	p=0.056
Private	43 (65.2%)	39 (49.4%)	
NCI affiliated institution N(%)			
Yes	13 (19.7%)	23 (29.1%)	p=0.19
No	53 (80.3%)	56 (70.9%)	
ACOG District N(%)			
District I (Atlantic provinces, CT, ME, MA, RI, VT)	3 (4.5%)	7 (8.9%)	p=0.91
District II (NY)	2 (3.0%)	2 (2.5%)	
District III (DE, NJ, PA)	3 (4.5%)	3 (3.8%)	
District IV (DC, GA, MD, NC, SC, VA, WV)	9 (13.6%)	11 (13.9%)	
District V (IN, KY, OH, MI)	5 (7.6%)	12 (15.2%)	
District VI (IL, IA, MN, NE, ND, SD, WI)	7 (10.6%)	7 (8.9%)	
District VII (AL, AR, KS, LA, MS, MO, OK, TN)	13 (19.7%)	14 (17.7%)	
District VIII (AK, AZ, CO, HI, ID, MT, NV, NM, OR, UT, WA, WY)	16 (24.2%)	16 (20.3%)	
District IX (CA)	2 (3.0%)	2 (2.5%)	
District XI (TX)	3 (4.5%)	1 (1.3%)	
District XII (FL)	3 (4.5%)	4 (5.1%)	
Insurance requested			
Yes	14 (21.2%)	8 (10.1%)	p=0.064
No	52 (78.8%)	71 (89.9%)	
*In 2023, a total of 18(23%) of offices said that both/either a male and/or female physician were available so N=9 instances of each gender were added to this demographic to reflect availability			

More academic practices offering appointments in 2023 (50.6%) compared to 2020 (34.8%), although this was not significant (p=0.056). Of these practices, less than 19.7% and 29.1% were affiliated with the NCI in 2020 and 2023, respectively. Offices from every ACOG district were sampled, with similar proportions from each district contacted during each calling period (p=0.91). The majority of offices did not request insurance prior to scheduling an appointment (78.8% in 2020, 89.9% in 2023, p=0.064), although the difference between years was not significant (p=0.064).

**Figure 2:**
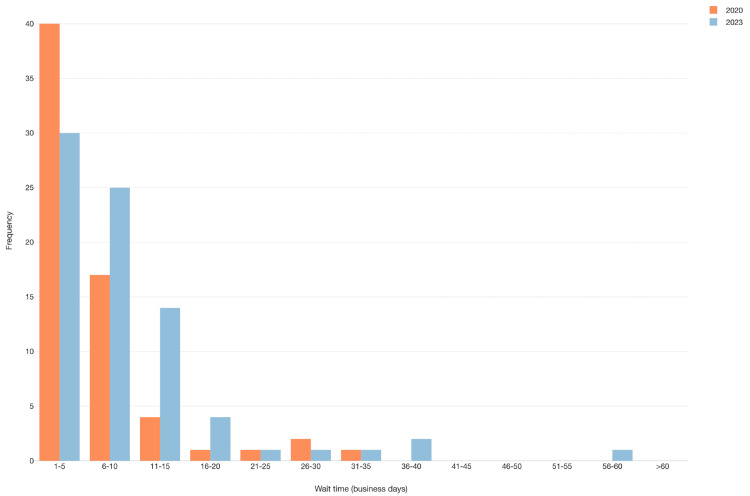
Histogram of wait times in business days by year. The x-axis depicts the category of wait time segmented five business days at a time. The y-axis depicts the frequency of wait times that occurred in each block. Orange bars display data from 2020 while blue bars display data from 2023

Using an unpaired t-test, mean wait times were significantly longer in 2023 compared to 2020 (10.3 business days (SD±9.2)) versus 6.6 business days ((SD±6.4), p=0.0067). The frequency of short wait times (one to five days) was higher in 2020 (n=40) compared to 2023 (n=30) (p=0.0066) (Figure 2). Overall, most offices had wait times of less than 15 days in 2020 (n=61, 92%) and 2023 (n=69, 87%). 

However, when using a multivariable model adjusting for NCI affiliation of the clinic, physicians’ gender, insurance, and clinic, year (2023 versus 2020) was not significantly associated with the wait time (p=0.27). The covariate of insurance was associated with wait time when adjusted for clinic, physicians’ gender, and insurance.

In further sub-analysis, we found that NCI-affiliated clinics had significantly shorter wait times than non-affiliates in 2023 (7.2 versus 11. 6 business days, p=0.0240) when adjusting for practice type, physician’s gender, and insurance. No such association was noted in a similar analysis for 2020 (6.1 versus 6.7 business days, p=0.8518). 

**Figure 3:**
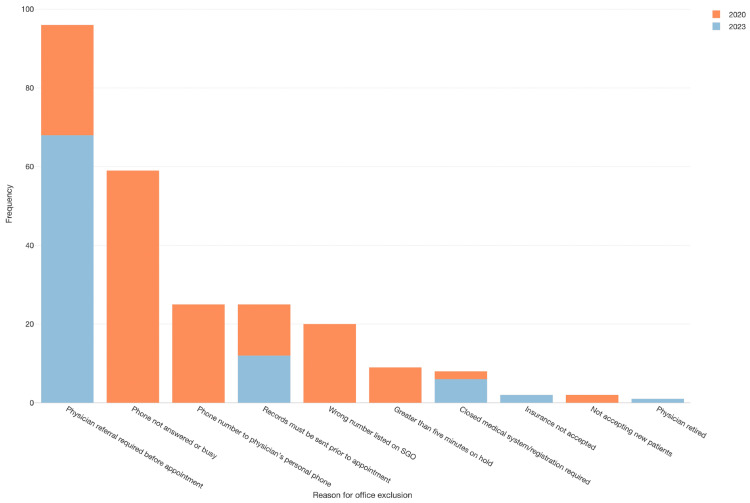
Stacked bar chart depicting reasons for office exclusion by year. The x-axis lists the reasons why a particular office was excluded from the wait time calculation and the y-axis denotes the frequency of each exclusion. Blue bars depict 2020 and orange bars depict 2023

Figures 1, 3 show all documented reasons why offices could not provide an appointment date after a contact attempt by a mystery caller during this study. The most common reason offices could not provide an appointment date was that the phone was not answered or had a busy signal during a call attempt (this includes having to leave a voicemail) (n=82, 35%) (Figure 3). The second most common reason was that a physician referral was required prior to scheduling an appointment (i.e., patients could not refer themselves) (n=56, 24%). There were additional issues related to the original SGO database including (1) a wrong number originally listed on the database (n=25,11%) and (2) a phone number for the physician's personal phone instead of a clinic number (n=25, 11%).

**Figure 4:**
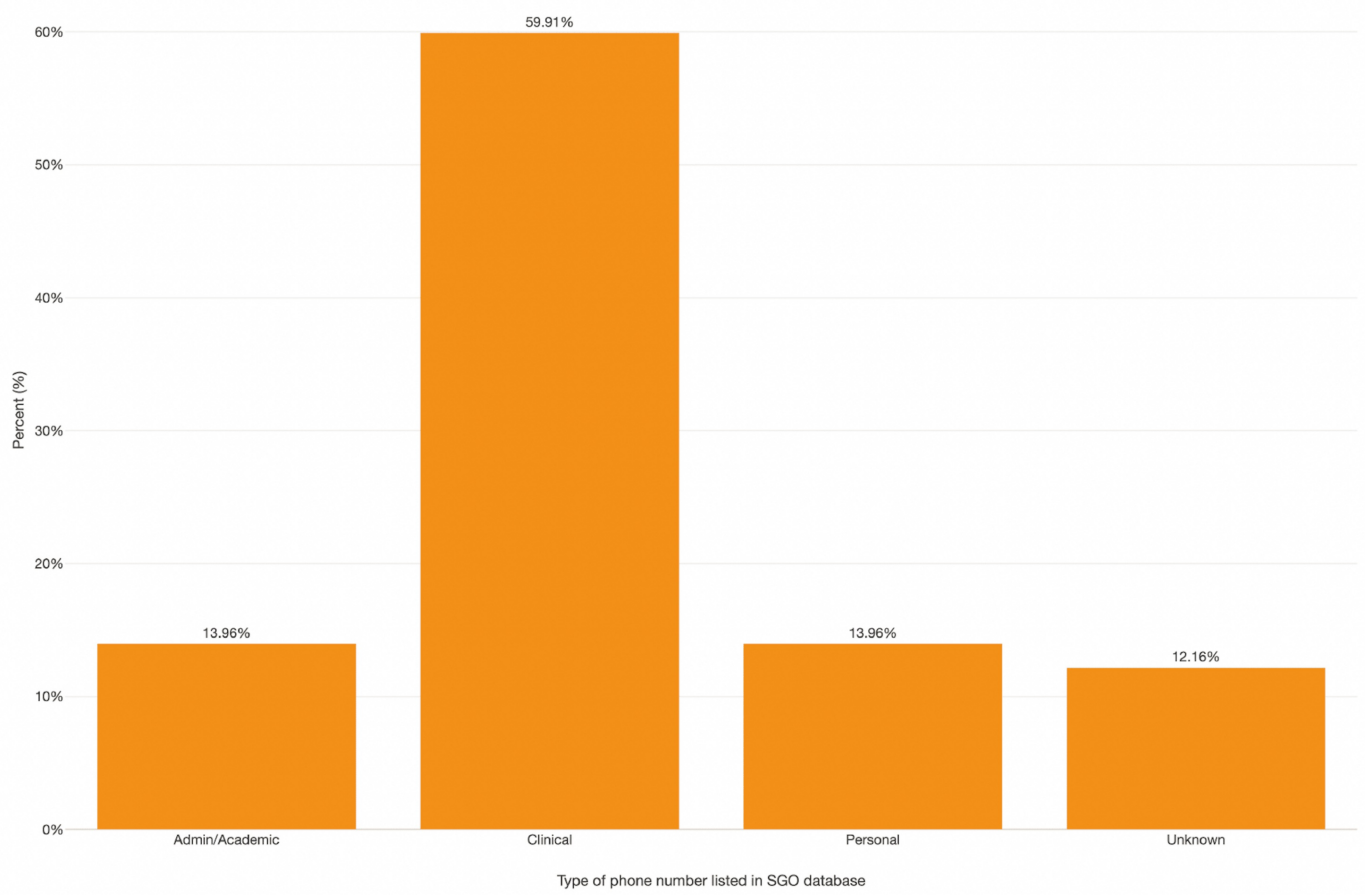
Bar chart depicting the type of number listed in the Society for Gynecologic Oncology database at the time of the study

Finally, the patient usefulness of the SGO database is shown in Figure 4. Approximately 60% of the numbers listed in the database were clinical, and thus, an appointment could be made by calling this number. The other 40% of the numbers were either administrative, personal, or unknown. Administrative numbers led to a research-focused office where an appointment could not be made.

## Discussion

To our knowledge, this is the first study to audit the impact of the COVID-19 pandemic on wait times for a first appointment with a gynecologic oncologist across the US. Our primary finding was that wait times were not significantly longer in 2023, after the height of the COVID-19 pandemic than in 2020.

Research conducted since 2020 has found that during the emergency phase of the pandemic, there were decreases and delays in cancer diagnoses [15-17]. While patients spoke up about delays in diagnosis and care during the pandemic, no quantitative research investigated the extent to which this was delayed specifically in gynecologic oncology care [18]. At a baseline, covert symptoms and lack of screening for ovarian cancer make diagnosis difficult in this patient population [4,19]. However, if ovarian cancer can be detected early, patients have better prognoses [19]. In the context of our findings, ovarian cancer patients are not facing excess delays in the post-emergency phase of the COVID-19 pandemic compared to the height of the pandemic. While a simple t-test found a significant difference in wait times during the pre- and post-emergency phase of COVID-19, our model which adjusted for paired clinic data, there was no difference in wait times. We interpreted these findings in two ways. First, we argue that this finding could reflect resilience in the healthcare system given that the backlog of patients from the emergent phase of the pandemic did not bottleneck the system and increase wait times in the post-emergent phase. One study surveying radiation oncologists prospectively during the pandemic found that 66% of providers reported surgical delays and 45% of providers experienced pauses or delays in any treatment type further supporting the existence of an early backlog in patient care [20]. However, our findings could also suggest that pandemic-related delays have persisted and not returned to pre-pandemic levels. It is difficult to determine exactly which interpretation might be more accurate, given the lack of nationwide audits of wait times for first appointments with a gynecologic oncologist prior to the pandemic. Importantly, our data can allow us to speculate the severity of wait times for gynecologic subspecialist care during and after the emergent phase of the pandemic. One study conducted in Jamaica in 2013 showed average wait times from referral to first subspecialist appointment were about two weeks, which reportedly met international standards [21]. We found that the average wait times in 2020 and 2023 for a first appointment were within the two-week range. Thus, even during the emergent phase of the pandemic, wait times met the international recommendation set by the European Board and College of Obstetrics and Gynecology which states “Women with suspected or proven cancer should have access to a gynae-oncology service within 2 weeks” [22].

Among clinics that provided appointment dates during the study, we observed a trend toward a greater proportion of academic clinics with appointment availability in 2023 compared to the early pandemic in 2020. We also observed that NCI-affiliated clinics had shorter wait times in 2023 than non-NCI-affiliated clinics. We speculate that this shift in appointment availability by practice type (academic versus private) and NCI-affiliation status may be in part due to academic centers/tertiary care centers caring for the majority of severe COVID-19 cases early in the pandemic [23,24]. Research exploring financial burdens and shifts in healthcare during the pandemic found that safety net hospitals faced the largest financial burdens during the pandemic [24]. A large contributor to the financial burden was academic centers/tertiary care centers performing fewer surgeries, including those for cancer patients, due to the system stress of caring for patients with COVID-19 [23]. With widespread vaccination, subsequent milder disease, and fewer hospitalizations, we hypothesize that academic centers were better equipped to take on new potential surgical cases in 2023, and thus were more likely to provide appointment times in 2023 than in 2020. Further, the finding that NCI centers had wait times of around one week (seven days) in 2023 is very similar to the findings of a pre-pandemic mystery caller study auditing 40 NCI-affiliated centers, which reported the majority of wait times to be greater than one week for the first appointment at a cancer center; however, this was not specific to gynecologic cancers [25]. One other possibility is that the increased contribution of benign operations among gynecologic oncologist cases might lead to increases in wait times for new patients, especially among private providers [26].

A primary clinical implication of our results is that close to 20% (n=45) of public-facing physician phone numbers did not lead to a clinical number that enabled appointment scheduling. When patient-facing data sources provide inaccurate information, it can delay patient information gathering, access to care, and time to first subspecialist appointment [27]. Locatelli et al. found that outdated phone registries led to patients delaying their care or seeking unscheduled medical help at the emergency room [27]. Given the high rate of inaccurate information in the directory and the potential for this to negatively impact care, we suggest that changes should be made at the level of national medical governing bodies to ensure that patient-facing information is accurate, thus decreasing any extra barriers to information and care. Busch and Kyanko suggested a federal standard for patient directories and enforcement of pre-existing laws [28]. Recently, ACOG retired its patient-facing physician directory, which may limit the amount of incorrect information provided to prospective patients/researchers, but also removes a critical resource. Patient-facing information, curation, and upkeep are arduous and often expensive tasks, but they should be a priority for national professional societies if they continue to make these resources publicly available. Further research could focus on assessing the reliability of OB/GYN public databases compared to other fields such as the database for the American College of Surgeons.

Finding the correct information for a provider was not the only burdensome aspect of receiving care we found and also scheduling a new patient can be difficult and time-consuming. Not only do many practices have long wait times to talk to a new patient coordinator, but many also require that the patient leave a message and receive a call back at another time. These barriers to scheduling a new patient or even to just inquiring about wait times provide extra barriers to patients who do not have consistent phone access or whose work impedes their ability to pick up a daytime phone call to coordinate scheduling. Matulis et al. suggest creativity and flexibility be incorporated into the scheduling process, potentially with the help of artificial intelligence to shape the process to the patient's needs [29]. In this way, a patient just requesting a wait time may be able to obtain it. At the same time, a patient who could have simple screening performed via telephone could also have this achieved before a face-to-face appointment, saving time for both patient and physician. Further, given the known staffing shortages, especially since the beginning of the pandemic, a focus can be made on retaining existing staff and hiring new scheduling staff as another way to improve overall patient care [30].

Limitation

There are several limitations to our study. First, obtaining physician phone numbers from a public database returned many incorrect numbers. As a result, our sample sizes were too small per state to make robust inferences about changes in state-based wait times for gynecologic oncology care longitudinally. However, this also helped us to identify a critical issue in the patient experience that can hopefully be addressed by professional societies and government agencies moving forward. Second, given the observational nature of the research, only correlation can be determined and not causality for the observed wait time differences. Third, our findings are specific to a female patient with a pelvic mass, and therefore cannot be confidently extended to predict wait times for a patient with a significantly different clinical presentation. It is possible that our small sample size with paired data impacted our ability to observe a difference in wait times between 2020 and 2023. Finally, calls were only made at two-time points. Measuring wait times in the fall of 2021 and 2022, in addition to 2020 and 2023, may have elucidated an increase and subsequent decrease in wait times that was masked by our long interval between measurements. Further studies with larger sample sizes are needed to corroborate our findings and explore factors that impact wait times for gynecologic oncology care.

## Conclusions

In this mystery caller audit of wait times for a first appointment with a gynecologic oncologist during and after the COVID-19 pandemic, overall wait times did not increase, emphasizing a previously undescribed resilience within the healthcare system. Further research is needed to understand, which factors manifested this resilience and to provide an example for fields that are still struggling with post-COVID-19 delays in treatment. Beyond this unexpected finding, our observations of incorrect patient-facing databases provide an actionable step toward eliminating barriers to care in gynecologic oncology. Effort on the part of healthcare organizations is needed to ensure that patient-facing information is updated to increase the ease with which patients can contact and schedule appointments in the field of gynecologic oncology.
